# Cytokine Storm Signature in Patients with Moderate and Severe COVID-19

**DOI:** 10.3390/ijms23168879

**Published:** 2022-08-10

**Authors:** Olga Kalinina, Alexey Golovkin, Ekaterina Zaikova, Arthur Aquino, Vadim Bezrukikh, Olesya Melnik, Elena Vasilieva, Tatiana Karonova, Igor Kudryavtsev, Evgeny Shlyakhto

**Affiliations:** 1 Almazov National Medical Research Centre, 197341 St. Petersburg, Russia; 2 Institute of Experimental Medicine, 197376 St. Petersburg, Russia

**Keywords:** COVID-19, cytokines, multiplex, chemokines, growth factors

## Abstract

Hypercytokinemia, found in SARS-CoV-2 infection, contributes to multiple organ dysfunctions with acute respiratory distress syndrome, shock etc. The aim of this study was to describe cytokine storm signatures in patients with acute COVID-19 and to investigate their influence on severity of the infection. Plasma levels of 47 cytokines were investigated in 73 patients with moderate and severe COVID-19 (41 and 32, respectively) and 11 healthy donors (HD). The most elevated levels comparing patients and the HD were observed for seven pro-inflammatory cytokines (IL-6, IL-8, IL-15, IL-18, IL-27, IFNγ, TNFα), three chemokines (GROα, IP-10, MIG), two anti-inflammatory cytokines (IL-1RA, IL-10), and two growth factors (G-CSF, M-CSF). The patients with severe disease had significantly higher levels of FGF-2/FGF-basic, IL-1β, and IL-7 compared to the HD. The two groups of patients differed from each other only based on the levels of EGF, eotaxin, and IL-12 p40. Pneumonia lung injury, characterized by computer tomography, positively correlated with levels of EGF, IP-10, MCP-3 levels and negatively with IL-12 p40. Pro-inflammatory factors including IL-6, TNFα, and IP-10 negatively correlated with the frequency of the circulating T-helper17-like cells (Th17-like) and follicular Th cells that are crucial to develop SARS-CoV-2-specific plasma cells and memory B cells. Obtained data on the cytokine levels illustrate their influence on progression and severity of COVID-19.

## 1. Introduction

Previous investigations have reported exuberant activation of innate and adaptive immune cells as well as coagulation systems in the blood of patients with COVID-19, accompanied by tissue inflammation in various locations [1,2,3,4]. Several studies reported that patients with moderate and severe COVID-19 presented an impaired T cell response and imbalance of main T-helper (Th) subsets [5,6,7] that could result in total dysfunctional cellular and humoral immunity in severe cases of COVID-19. Furthermore, the increased and uncontrolled systemic production of pro- and anti-inflammatory cytokines has been seen to simultaneously contribute to the pathophysiology of severe COVID-19 and acute respiratory distress syndrome [8,9]. Hypercytokinemia, which is also referred to as ‘cytokine storm’, was found in SARS-CoV-2 infection and was thought to contribute to multiple organ dysfunction with acute respiratory distress syndrome, shock, and renal failure [10]. During the acute phase of SARS-CoV-2 infection, serum concentrations of pro-inflammatory IL-6, IL-8, IL-2R, TNF-alpha, and anti-inflammatory IL-10 cytokines strongly correlated with disease outcome and were increased in patients with severe disease [11]. Also, cytokine production in patients with COVID-19 was found to be imbalanced with regard to controlling immune-mediated tissue damage versus wound healing and tissue repair response [12,13]. Secondary hemophagocytic lymphohistiocytosis (macrophage activation syndrome), a hyperinflammatory syndrome characterized by the release of cytokines, cytopenia, and multi-organ failure, may develop in addition to high levels of cytokines [14].

Currently, a wide spectrum of pro- and anti-inflammatory cytokines/chemokines is known that can trigger or effect various pathologic processes and cause long-term consequences. Thus, the aim of this study was to describe cytokine storm signature in patients with acute COVID-19 and to investigate their influence on severity of the infection.

## 2. Results

**Figure 1:**
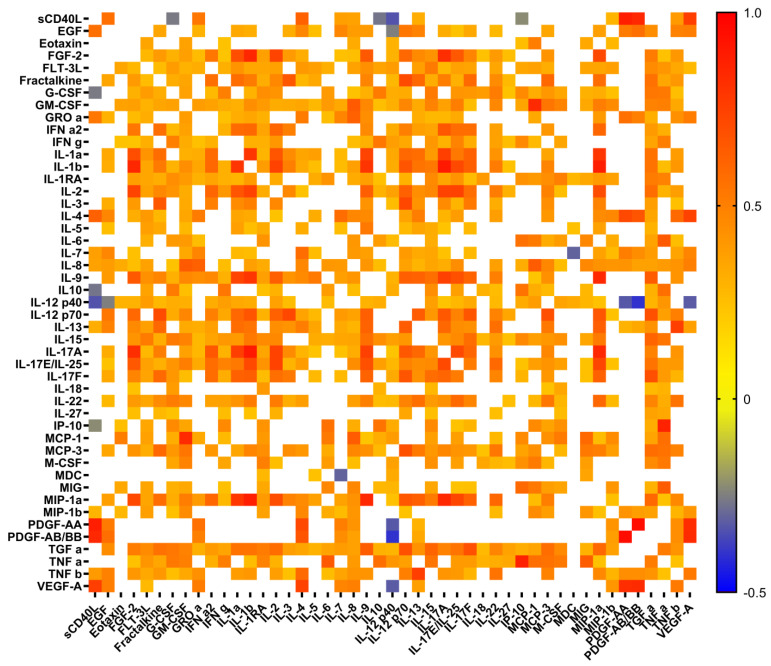
Heat map of correlation between cytokine levels in plasma samples from patients with COVID-19. Only significant correlations are presented. Color scale bars show a range of correlation coefficients (r). The red color represents a high positive correlation, decreasing to the blue color bar, which represents a negative correlation.

The levels of 22 cytokines in both groups of patients were significantly higher than those in healthy donors (HD). The most elevated levels were observed for seven pro-inflammatory cytokines (IL-6, IL-8, IL-15, IL-18, IL-27, IFNγ, TNFα), three chemokines (GROα, interferon gamma-induced protein 10 (IP-10), monokine induced by gamma interferon (MIG)), two anti-inflammatory cytokines (IL-1RA, IL-10), and two growth factors (G-CSF, M-CSF) (Table 1). The patients with severe disease had significantly higher levels of basic fibroblast growth factor (FGF-2/FGF-basic, endothelial factors involved in regulation of vascular function), IL-1β, and IL-7 compared to the HDs. While the patients with moderate disease had increased levels of macrophage-derived chemokine (MDC) and macrophage inflammatory protein (MIP-1β), they exhibited decreased levels of sCD40L and both platelet-derived growth factors (PDGF-AA and PDGF-AB/BB) compared to HDs. Interestingly, the two groups of patients differed from each other only based on the levels of epidermal growth factor (EGF), eotaxin, and IL-12 p40. The levels of vascular endothelial growth factor (VEGF-A), also involved in regulation of vascular function, as well as chemokine FLT-3 ligand, one of the markers of endothelial injury, were not significantly different in both studied groups. Correlation analysis revealed a multiple network of cytokine interactions; in the majority of cases, a positive correlation was observed, while for only a few cytokines, it was negative (in particular, IL12 p40 vs. sCD40L, IL12 p40 vs. EGF, IL12 p40 vs. PDGF-AB/BB) (Figure 1).

**Figure 2:**
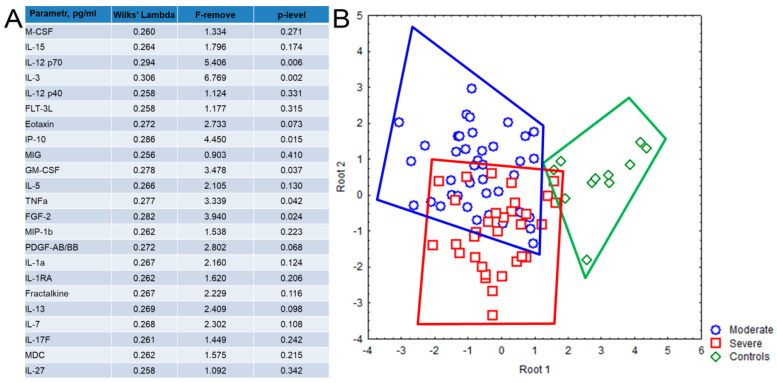
Discriminant analysis based on the cytokine levels in plasma samples, obtained from patients with COVID-19 and healthy donors, consisted of 23 steps and 23 variants in the model with Wilks’ lambda = 0.249, approx. F (46.118) = 2.572, *p* < 0.001. (**A**) The list of 23 cytokines with high grouping and discriminant value included in the model. (**B**) Dot plot shows the clusters formed by the patients with moderate (blue color) and severe (red color) disease and healthy donors (green color). Each dot represents one person.

According to the discriminant analysis (Figure 2), a high degree of discrimination was established for 23 out of 47 studied cytokine/chemokines/growth factors between patients with COVID-19 and healthy donors. The strongest discriminating factors were IL-12 (p70), IL-3, IP-10, FGF-2/FGF-basic, GM-CSF, TNFα, PDGF-AB/BB (Figure 2A). The patients from both groups and healthy donors form tighter but separate clusters based on their cytokine profiles, whereas patient’s groups clusters partially overlap (Figure 2B).

**Figure 3:**
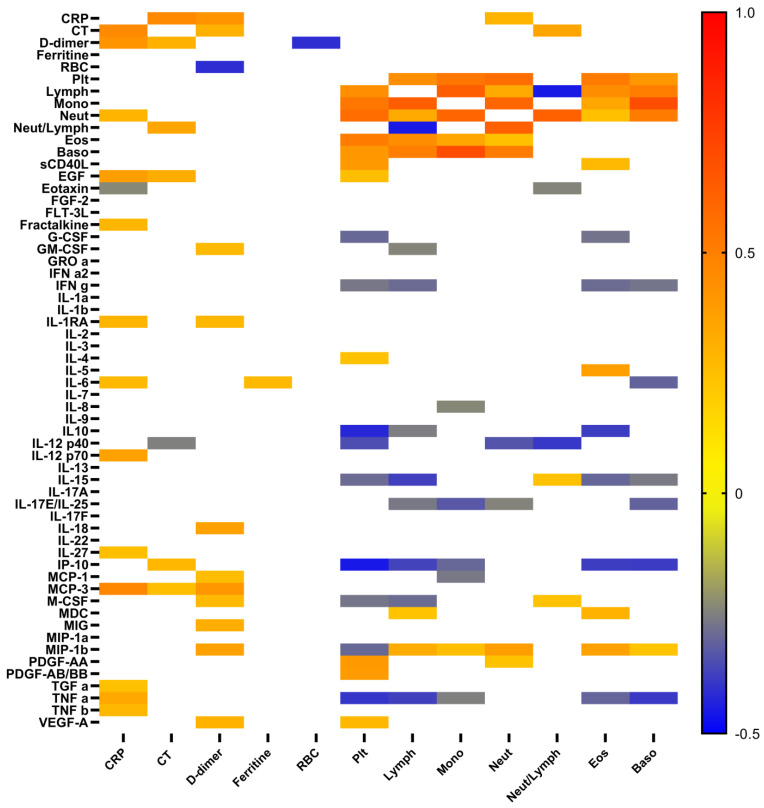
Heat map of correlations between cytokine levels, and some conventional clinical and laboratory markers in patients with COVID-19. Color scale bar show a range of correlation coefficient (r). The red color represents a high positive correlation, decreasing to the blue color bar, which represents a negative correlation.

To analyze possible correlations between cytokine levels and the degree of lung damage, as well as levels of ferritin, C-reactive protein, and D-dimer, which are associated with the severity and outcome of the disease, we performed appropriate correlation analysis (Figure 3). As expected, increased levels of C-reactive protein (CRP) positively correlated with elevated levels of TNF α, TNF β, TGF α, MCP-3, IL-27, IL-12 p40, IL-6, IL-1RA, fractalkine, and EGF, but negatively with eotaxin. In contrast, the levels of the D-dimer had a positive correlation with chemokines MIP-1α, MIG, MCP-3, MCP-1, pro-inflammatory cytokine IL-18, anti-inflammatory cytokine IL-1RA, and various growth factors (VEGF-A, M-CSF, and GM-CSF). The levels of platelets had positive correlations with both platelet-derived growth factors (PDGF-AA and PDGF-AA/AB), VEGF-A, EGF, sCD40L, and IL-4, but negative correlations with IL-10, IL-12p40, IL-15, IP-10, M-CSF, MIP-1b, TNF α, IFNγ, and G-CSF. The elevated ferritin level was associated only with pro-inflammatory cytokine IL-6. As expected, pneumonia lung injury characterized by computer tomography (CT) positively correlated with levels of CRP, D-dimer, and neutrophils/lymphocytes ratio. In contrast, positive correlations with EGF, IP-10, MCP-3 levels and negative with IL-12 p40 were quite unexpected.

**Figure 4:**
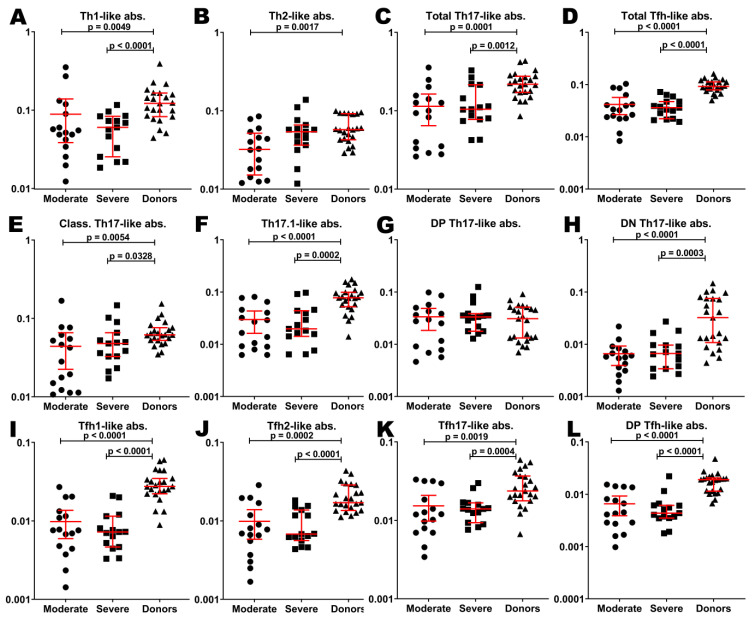
Basic Th cell subsets in patients with COVID-19 and in healthy donors. Absolute counts of Th1-like (**A**), Th2-like (**B**), total Th17-like (**C**), total Tfh-like (**D**), classical Th17-like (**E**), Th17.1-like (**F**), double-positive Th17-like (**G**), double-negative TH17-like (**H**), Tfh1-like (**I**), Tfh2-like (**J**), Tfh17-like (**K**), double-positive Tfh-like (**L**) cells. Long red lines demonstrate median value, short red lines demonstrate interquartile range.

To evaluate the relationship between cytokine profiles and circulating Th-cell patterns depending on the severity of the course of the disease, we analyzed the frequencies of peripheral blood Th-cell subsets. Probably, because of the lymphopenia in patients with COVID-19, main Th-subsets were decreased comparing to healthy donors. In particular, absolute counts of Th1-like (0.05 (0.04;0.10) cells/µL), Th2-like (0.03 (0.02;0.05) cells/µL), total Th17-like (0.09 (0.03;0.14) cells/µL), and total follicular T-helpers (Tfh)-like (0.03 (0.02;0.05) cells/µL) cells in patients with moderate COVID-19 were lower comparing to HD (0.12 (0.08;0.17), 0.05 (0.04;0.09), 0.21 (0.17;0.27), and 0.09 (0.08;0.11) cells/µL, respectively) (Figure 4A–D). All mentioned Th-subsets except Th2-like cells were also significantly decreased in patients with severe infection (0.06 (0.02;0.08) cells/µL, 0.05 (0.03;0.06) cells/µL, 0.10 (0.07;0.21) cells/µL, and 0.03 (0.02;0.05) cells/µL of Th1-like, Th2-like, total Th17-like, and total Tfh-like cells, respectively).

Only double-positive (DP) Th17-like subsets in both groups of patients (0.028 (0.008;0.046) cells/µL and 0.033 (0.018;0.038) cells/µL for moderate and severe COVID-19, respectively) were equal to those in HD (0.030 (0.013;0.051) cells/µL) (Figure 4G). Classical Th17-like cells, Th17.1-like cells, and double-negative (DN) Th17-like cells were decreased in patients with moderate infection (0.03 (0.01;0.05) cells/µL, 0.02 (0.01;0.04) cells/µL, and 0.005 (0.003;0.007) cells/µL, respectively) compared to donors (0.06 (0.05;0.07) cells/µL, 0.08 (0.05;0.10) cells/µL, and 0.032 (0.010;0.075) cells/µL, respectively). Indicated subpopulations were also decreased in patients with severe COVID-19 (0.04 (0.03;0.06) cells/µL, 0.02 (0.01;0.04) cells/µL, and 0.006 (0.003;0.009) cells/µL, respectively) (Figure 4E,F,H).

All Tfh-cell subsets in both groups of patients were decreased compared to HD. In particular, Tfh1-like cells (0.007 (0.004;0.012) cells/µL, 0.007 (0.004;0.011) cells/µL, and 0.027 (0.022;0.034) cells/µL in moderate, severe infection and HD, respectively), Tfh2-like cells (0.007 (0.004;0.014) cells/µL, 0.006 (0.005;0.013) cells/µL, and 0.017 (0.013;0.029) cells/µL, respectively), Tfh17-like cells (0.011 (0.007;0.024) cells/µL, 0.014 (0.009;0.016) cells/µL, and 0.023 (0.017;0.036) cells/µL, respectively), and DP Tfh-like cells (0.004 (0.002;0.010) cells/µL, 0.004 (0.003;0.006) cells/µL, and 0.019 (0.011;0.020) cells/µL, respectively) were decreased in the case of COVID-19 (Figure 4I–L).

Of note, there were no significant differences in absolute counts of investigated T-cell subsets in both groups of patients.

**Figure 5:**
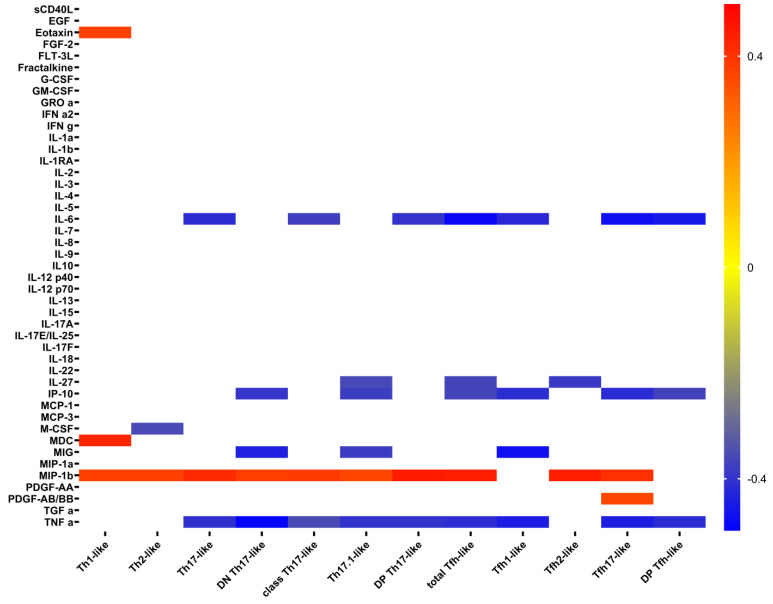
Heat map of correlations between cytokine levels and absolute count of Th-cell subsets in patients with COVID-19. Color scale bar shows a range of correlation coefficients (r). The red color represents a high positive correlation, decreasing to the blue color bar, which represents a negative correlation.

Correlation analysis demonstrated some unexpected relationships between cytokines levels and Th cell subsets. Surprisingly, there were significant negative correlations between IL6 levels and total Th17-like, classical Th17-like, DP Th17-like, total Tfh-like, Tfh1-like, Tfh17-like, and DP Tfh-like cells (Figure 5). Also, there were unexpected positive correlations between MIP-1b and almost all investigated T-cell subsets. Meanwhile, positive correlations of IP-10 were expected.

## 3. Discussion

COVID-19 is an infectious disease that causes an imbalance in the immune system and initiates an inflammatory cytokine storm, but long-term loss of control in cytokine network may lead to unintended consequences [15] and may result in significant changes in the composition of circulating immune cells, which persist for a long time [16]. Since the emergence of SARS-CoV-2, numerous studies have described abnormal levels of certain cytokines and chemokines, an uncontrolled increase in the production of pro-inflammatory IL-6, IL-8, IL-17 CCL2/MCP-1 and anti-inflammatory IL-10, CXCL10/IP-10 cytokines and an effective depletion of the antiviral defenses of innate immunity in patients with acute COVID-19 (reviewed in [17,18,19]). In this study, multiplex analysis revealed that 22 out of 47 analyzed cytokines, including pro-(such as IL-6, IL-18, TNF α, IFN α2) and anti-inflammatory (such as IL-1RA, IL-10, and IL-27) cytokines, multiple pro-inflammatory chemokines (IP-10, MCP-1, MIP-1a, MIG, IL-8, eotaxin, GROa), mediators of cell proliferation and differentiation (M-CSF, G-CSF, EGF, TGF α, IL-3, IL-15), as well as Th1 (IFN γ), Th2 (IL-5), Th17 (IL-17F) cells were significantly elevated in both groups of patients with COVID-19 compared to HDs. These results indicated a serious immune disorder that previously was reported in other pathogenic coronaviruses, including Middle East respiratory syndrome coronavirus (MERS) [20] and severe acute respiratory syndrome coronavirus [21].

### 3.1. Dysbalanced Cytokine Levels in Patients with Acute COVID-19

The elevated levels of TNF α, IL-1RA, IL-6, IL-8, IL-10, IP-10, G-CSF and FGF-2 in both patient groups with COVID-19 in our study coincided with previously published studies [17,18,19,22,23]. However, some cytokines, such as MIP-1b (CCL4), IL-1β, PDGF-AA, PDGF-AB-BB, and soluble CD40L, yielded controversial results. In particular, in our cohort, the plasma levels of two multiple pro-inflammatory chemokines, MIP-1b and MDC, were increased in patients with moderate COVID-19 compared to HDs, but had no differences between patients with severe COVID-19 and HDs. Previously, it was found that the MIP-1b was inversely associated with COVID-19, indicating a protective role of MIP-1b in COVID-19 vulnerability and hospitalization [24], whereas MDC concentrations were decreased in mild to severe/critical patients, and the lowest levels of MDC were detected during the late phases of disease in severe/critical patients [25].

We determined a significant reduction in levels of PDGF-AA, PDGF-AB-BB, and soluble CD40L in patients with moderate COVID-19 compared to HDs and found no difference between severe patients and HDs. Their concentrations positively correlated with the platelet levels in blood samples (Figure 3). Several studies have reported a significant increase in the levels of these growth factors, which are known to mediate vasculitis and vascular remodeling due to pro-angiogenic properties involved in angiogenesis and vascular remodeling in infected patients [22,23]. Petrey et al. indicated that levels of PDGF-AA, PDGF-AB-BB, sCD40L, FGF, and IP-10 were doubled among COVID-19 patients relative to healthy controls [22]. Similarly, sCD40L was increased in both bronchoalveolar lavage (BALF) fluid and in plasma from totally 22 patients compared to controls [26], but conversely, PDGF-AA and PDGF-BB levels were elevated in BALF yet not in serum of patients with COVID-19 [26].

Moreover, in our cohort group, IL-1β, IL-7 and FGF-2 elevated in samples from patients with severe, but not with moderate COVID-19 compared to HDs. According to previously published data, IL-1β was not always increased in severe COVID-19 cases [25,27], but was significantly lower in asymptomatic cases than in symptomatic patients, suggesting IL-1β levels may be predictors of clinical symptoms [28]. In contrast, FGF-2 expression was found to be upregulated in lungs in patients who died from COVID-19 [29]. Interestingly, the levels of IL-1β and FGF-2 together with MCP-1, MIP-1α, and IP-10 were found to be related to thrombosis [30]. Elevated IL-7 levels were found to be associated with the severity of COVID-19 [28] and admission to intensive care units [31].

### 3.2. Differences between Cytokine Levels in Groups of Patients According to the Severity of the Infection

Thus, the question of interest is if patients have a special cytokine status or cytokine storm signature depending on the severity of the disease. Performed discriminant analysis proved the hypothesis about multiple involvements of cytokine networks in storming in patients with COVID-19 and about the specificity of cytokine storm profile according to severity of the infection. The estimated model, containing 23 cytokines, with Wilks’ lambda equal to 0.249, demonstrated that cytokine storm in patients with COVID-19 was very specific. Meanwhile, the severity of SARS-CoV-2 infection affects the nature of the cytokine storm.

Particular attention was paid to the analysis of biomarkers, the level of which does not match in patients with moderate and severe course of disease. In this study, the patients with moderate and severe COVID-19 significantly differed from each other based on the levels of three cytokines eotaxin, IL-12p40, and EGF.

We found that the levels of eotaxin, which is involved in recruitment of eosinophils to diverse tissues, were increased in patients with moderate COVID-19 compared to patients with severe illness. Previously, it was shown that eosinopenia could serve as an important predictor of disease severity, and alterations in eosinophil frequency significantly correlated with disease progression in patients with COVID-19 [32]. Moreover, eosinophil counts were significantly lower in patients with critical disease when compared to those with moderate and severe cases, and non-survivors [33]. Furthermore, eosinophil counts significantly and inversely correlated with some conventional clinical and laboratory markers, including CRP, procalcitonin, and ferritin. Negative correlation between eotaxin and CRP levels in our patients indicated that progressive eotaxin decline was linked with inflammation and worsening of viral infection. However, Zhang et al. showed that abnormally elevated eotaxin levels (together with MCP-1 and IP-10) may help to determine the severity of SARS-CoV-2 infection [34]. However, some studies did not revealed significant differences in the concentration of eotaxin in patients with COVID-19 [28].

In our cohort groups, the plasma levels of IL-12p40 were decreased in patients with severe COVID-19 compared to patients with moderate disease. Currently, IL-12p40 is considered not only as an intrinsically functional cytokine, but also a cytokine that may play a pivotal role in initiating an immune response by stimulating macrophage chemotaxis and promoting the migration of activated dendritic cells [35]. In numerous articles, the decrease in circulating monocytes and dendritic cells in response to acute SARS-CoV-2 infection was reported [36,37,38]. Moreover, dendritic cells, obtained from patients with COVID-19, expressed significantly less CD80, CD86, CCR7, and HLA-DR in response to in vitro stimulation compared to those from HDs [39]. Meanwhile, reduced expression of HLA-DR on classical monocytes has characterized severe cases of SARS-CoV-2 infection and could correlate with inflammation response, including IL-6 overproduction [40,41].

Interestingly, we found that IL-12p40 levels negatively correlated with CT data, whereas the positive correlations were observed with variety of upregulated pro-inflammatory cytokines and chemokines, indicating dysbalanced immune response and the important role of this molecule as a potential marker of disease severity (Figure 2 and Figure 3). Furthermore, Ling et al. also revealed decreased levels of circulating IL-12p40 in severe cases of COVID-19 during the late phase of disease [25].

We determined elevated levels of EGF in patients with severe COVID-19 compared to patients with moderate disease. This cytokine is known to be a soluble growth factor that upregulates the proliferation activity of different types of cells, especially fibroblasts and epithelial cells, and could act as mediator of wound-healing and tissue repair. We also found that plasma concentration of EGF positively correlated with CRP levels and CT data as well as with various cytokines, including pro-inflammatory IL-1α and IL-1β, chemokines GROa (CXCL1), fractalkine (CX3CL1), IL-8, MCP3 (CCL7), and MIP-1a (CCL3), as well as Th2 (IL-4, IL-5, and IL-13) and Th17 (IL-17A, IL-17F, IL-17E/IL-25, and IL-22) cytokines. Thus, plasma EGF could be closely linked with inflammatory response of innate and adaptive immunity and reflect the severity of COVID-19. Recently, it was shown that EGF concentration was higher in plasma from patients with critical COVID-19 [42]. Furthermore, EGF was considered to be a marker of repair after either muscle or kidney tissue injury in patients with severe COVID-19 [43].

### 3.3. Relationships of Cytokines Levels and Levels of Immune Cells

The majority of patients infected with SARS-CoV-2 showed abnormal and reduced leukocyte counts and lymphopenia associated with the progression of viral infection [44], accompanied with uncontrolled cytokine production [10]. Furthermore, lymphopenia was usually accompanied by an increased number of neutrophils and high concentrations of pro- and anti-inflammatory cytokines and chemokines [23].

We analyzed relationships between cytokine profile and main leukocytes subsets. We observed negative correlations between lymphocyte counts and main pro-inflammatory (TNF α, IFN γ, IP-10, members of IL-17 family cytokines, GM-CSF, M-CSF, and regulatory (IL-10 and IL-15)) plasma factors. Circulating neutrophils negatively correlated with IL-17E/IL-25 and IL-12p40 concentrations, but positively with CRP levels. Finally, peripheral blood counts of eosinophils and basophils negatively correlated with pro-inflammatory TNF α, IP-10, IFN γ, and IL-15. Interestingly, IL-15 plays an important role in antiviral immune response via activation of natural killer (NK) cells and CD8+ T cells, stimulating their proliferation, as well as enhancing their effector capabilities [45], whereas prolonged exposure of NK cells to the circulating IL-15 might be responsible for the reduction of their cytolytic activity [46]. Thus, IL-15 levels were consistently higher throughout the hospitalization in patients who died versus those who recovered, suggesting that these biomarkers may provide an early warning of eventual disease outcome [47]. Similarly, IL-15 has positively correlated with longer hospitalization and more severe disease [48]. Thus, increased IL-15 levels and the associations of IL-15 with other clinical parameters could represent its signature in patients with severe COVID-19.

We noticed that circulating basophil frequencies negatively correlated with IL-17E/IL-25 and IL-6 levels. It is known that IL-25 is produced by epithelial tuft cells (and possibly other cells) in response to cell damage [49], whereas low absolute and relative basophil counts have been associated with a high risk of developing severe COVID-19 [50], SARS [51], and MERS [52] coronavirus infections.

Surprisingly, we found negative correlations between absolute numbers of Th17 cell subsets and main pro-inflammatory factors IL-6, IP-10, and TNF α. It is known that overproduction of IL-6 promoted pulmonary fibrosis and respiratory and multi-organ failure and has also been linked with pneumonia [53]. Also, IL-6 was found to be elevated in patients with COVID-19 and related to a poor prognosis [10]. Similarly, TNF α levels in patients with COVID-19 were increased, and they were closely associated with increased disease severity [54]. Furthermore, increased serum levels of IP-10 were associated with the severity of COVID-19 disease and could be related to the risk of death [55]. Previously, we have found that patients with severe COVID-19 had decreased frequencies of Th17-like cells in circulation compared to healthy controls and patients with moderate COVID-19 [7]. In agreement with our data, De Biasi et al. and Gutiérrez-Bautista et al. noticed that patients with COVID-19 displayed low frequencies of CD4+ T cell that expressed different Th17 cell-surface antigens [56,57]. Moreover, CD4+ T cells, co-expressing CCR6 and IL-17A, were detected in lung tissues of patients with COVID-19. Also, high concentrations of IL-6 and the main Th17 effector cytokines—IL-17A, GM-CSF, and IFNγ—were found in BALF [58]. Thus, we can assume that high levels of pro-inflammatory IL-6, IP-10, and TNF α could promote pulmonary inflammation, followed by Th17 and neutrophil migration to the lungs, where they could secrete different cytokines and chemokines that provide inflammation and tissue damage through various effector mechanisms.

Interestingly, we also have shown negative correlations between absolute numbers of follicular Th cell subsets (including Tfh1-like, Tfh17-like, and DP Tfh-like) and pro-inflammatory IL-6, IP-10, and TNF α. Tfh cells play a critical role in specific humoral immune response by controlling B cell maturation and differentiation in B-dependent areas of peripheral lymphoid tissue [59]. The capacity of Tfh1 cells to promote antibody production and to stimulate ‘naïve’ B cell differentiation to effector plasma cells was very limited, pointing to regulatory properties of this Tfh cell subset linked with suppression of the immune response [60]. However, Tfh1 cells play an important role in humoral antiviral immune response by efficiently inducing memory B cells and up-regulation of in vitro influenza-specific IgG production, while in vivo, the emergence of blood Tfh1 correlated with the development of protective anti-influenza antibody responses generated by memory B cells in response to vaccination [61,62]. Furthermore, ‘naïve’ B cells appeared to be stimulated by cTfh17 that were able to induce IgA production by plasma cells in vitro [60]. Moreover, in patients with COVID-19, the majority of SARS-CoV-2-specific Tfh cells had Tfh17 CCR6+CXCR3– phenotype [63]. Our previous analysis of Tfh cell subsets in patients with COVID-19 revealed a substantial shift toward a “pro-inflammatory” Tfh17 phenotype that was linked with COVID-19 severity [7]. Thus, we found that the increased concentrations of pro-inflammatory IL-6, IP-10, and TNF α were closely linked with the imbalance of Tfh cell subsets. These data indicate that cytokine and chemokine dysregulation may affect germinal center reactions and disturb Tfh differentiation. Therefore, the proper balance between regulatory Tfh1 cells and pro-inflammatory Tfh17 cells is important for the development of SARS-CoV-2-specific plasma cells and memory B cells.

Limitations: Most of the patients were over 50 years of age. Due to the limited number of people over 50 years of age with no signs of any chronic disease, the formation of a group of healthy donors was problematic and led to significant differences in the median age of the compared groups.

## 4. Materials and Methods

A total of 73 patients with COVID-19 who were admitted into the infectious disease department at Almazov National Medical Research Center (St. Petersburg, Russia) within 5–7 days after illness onset and 11 apparently healthy donors (10 men and 1 woman; median age 40 (32;47)) were included in the study. All samples were obtained for scientific research according to the Helsinki Declaration and approved by the local ethics committee (protocol no. 2209-20; 21 September 2020).

The COVID-19 was diagnosed as described previously [7,64] and was based on clinical data, typical chest computer tomography results, molecular and serological (routine testing for the presence of SARS-CoV-2 RNA in nasopharyngeal swabs (“SARS-CoV-2/SARS-CoV” PCR detection kit (DNA-technology TC, Moscow, Russia)), and anti-SARS-CoV-2 IgM/IgG in plasma samples (“DS-ELISA-anti-SARS-CoV-2” detection kit (RPC Diagnostic Systems, Nizhny Novgorod, Russia)), respectively). 78% of patients had positive PCR tests, whereas others did not have detectable levels of SARS-CoV-2 RNA in throat swabs but were positive for the anti-SARS-CoV-2 IgM/IgG. A total of 41 patients (19 men and 22 women; median age 61.0 (52.0;69.0)) out of 73 had moderate COVID-19 (SpO2 > 93%, respiratory rate <22/min, qSOFA < 2) and 32 (14 men and 18 women; median age: 59.0 (49.5;65.0) had severe COVID-19 (SpO2 < 93%, respiratory rate > 30/min, PaO2/FiO2 ≤ 300 mm Hg, qSOFA > 2). All apparently healthy donors were negative for the presence of anti-SARS-CoV-2 IgM/IgG.

Patients with moderate and severe COVID-19 had such comorbidities as arterial hypertension, diabetes mellitus, and ischemic heart disease, but their distribution inside groups was the same. Both groups of patients had significantly lower levels of red blood cells (RBC), hematocrit (Ht), lymphocytes, eosinophils, basophils, and neutrophils/lymphocytes ratio compared to healthy donors (Table 2). The CT results showed that patients with severe infection had significantly higher lung damage (*p* < 0.01), CRP (*p* < 0.05), and lactate dehydrogenase (LDH) (*p* < 0.001) levels. Further, patients with severe infection showed decreased monocyte counts compared to the HD.

Peripheral blood samples were collected before treatment initiation into vacuum test tubes containing K3-EDTA anticoagulant. Blood samples were centrifuged at 1000× *g* for 30 min, followed by collecting of plasma to cryotubes and stored at −40 °C until usage.

The levels of 47 cytokines/chemokines/growth factors in plasma samples were assessed by multiplex analysis performed on the fluorescently labeled magnetic microsphere beads using the MILLIPLEX^®^ MAP Human Cytokine/Chemokine/Growth Factor Panel A (HCYTA-60K-PX48, MilliporeSigma, Burlington, MA, USA) according to the manufacturer’s instructions on the Luminex MAGPIX^®^ (RUO) Instrument (Luminex, Austin, TX, USA). Briefly, 25 µL of plasma samples and fluorescently labeled magnetic microsphere beads were added into the appropriate wells and incubated with agitation on a plate shaker overnight at +4 °C in the dark. After washing, the antibodies for detection were added into wells and incubated with agitation 1 h at room temperature in the dark following by incubation with streptavidin–phycoerythrin in the same conditions. The standard samples were prepared according to the manufacturer’s recommendations. The calibration curve was created for each analyte separately. All the data were generated with xPONENT software and analyzed with Milliplex Analyst 5.1 Flex software.

The studied analytes were interleukins IL-1α, IL-1β, IL-1 receptor antagonist (IL-1Ra), IL-2, IL-3, IL-4, IL-5, IL-6, IL-7, IL-9, IL-10, IL-12 (p40), IL-12 (p70), IL-13, IL-15, IL-17A/CTLA8, IL-17-E/IL-25, IL-17F, IL-18, IL-22, IL-27; chemokines CCL2/MCP-1, CCL3/MIP-1α, CCL4/MIP-1β, CCL7/MCP-3, CCL11/eotaxin, CCL22/MDC, CXCL1/GROα, CXCL8/IL-8, CXCL9/MIG, CXCL10/IP-10, CX3CL1/fractalkine; growth factors EGF, FGF-2/FGF-basic, Flt3 ligand, G-CSF, M-CSF, GM-CSF, PDGF-AA, PDGF-AB/BB, TGF-α, VEGF-A; and pro-inflammatory cytokines IFNα2, IFNγ, TNFα, TNFβ/lymphotoxin-α(LTA) and soluble form of CD40L (sCD40L).

The Th-cell subsets were investigated using multicolor flow cytometry. For this purpose, blood samples were collected into vacuum test tubes containing K3-EDTA anticoagulant. A clinical blood analysis was performed using a Cell-DYN Ruby Hematology Analyzer (Abbott, Abbot Park, IL, USA). The immunophenotyping procedure was performed less than 6 h after blood collection using a CytoFlex S Flow Cytometer (Beckman Coulter, Indianapolis, IN, USA). The staining protocol, phenotyping procedure, gating strategy, and analysis were described previously in detail [7]. Briefly, a whole peripheral blood (100 µL) sample was stained using FITC-labeled mouse anti-human CD45RA (clone ALB11, cat. IM0584U, Beckman Coulter, Indianapolis, IN, USA), PE-labelled mouse anti-human CD62L (clone DREG56, cat. IM2214U, Beckman Coulter, Indianapolis, IN, USA), PerCP/Cy5.5-labeled mouse anti-human CXCR5 (CD185, clone J252D4, cat. 356910, BioLegend, Inc., San Diego, CA, USA), PE/Cy7-labeled mouse anti-human CCR6 (CD196, clone G034E3, cat 353418, BioLegend, Inc., San Diego, CA, USA), APC-labeled mouse anti-human CXCR3 (CD183, clone G025H7, cat. 353708, BioLegend, Inc., San Diego, CA, USA), APC-Alexa Fluor 750-labeled mouse anti-human CD3 (clone UCHT1, cat. A94680, Beckman Coulter, Indianapolis, IN, USA), Pacific-Blue-labeled mouse anti-human CD4 (clone 13B8.2, cat. B49197, Beckman Coulter, Indianapolis, IN, USA), and Brilliant Violet 510-labeled mouse anti-human CCR4 (CD194, clone L291H4, cat. 359416, BioLegend, Inc., San Diego, CA USA). Blood samples were stained with antibodies at room temperature for 15 min in the dark. Then, erythrocytes were lysed by adding 1 mL of VersaLyse Lysing Solution (Beckman Coulter, Inc., Indianapolis, IN, USA) with 25 µL of IOTest 3 Fixative Solution (Beckman Coulter, Inc., Indianapolis, IN, USA) in the dark at room temperature for 15 min. Next, all samples were washed (330× *g* for 8 min) twice with sterile PBS supplemented with 2% of fetal calf serum (FCS) (Sigma-Aldrich Co., Saint Louis, MO, USA), resuspended in 500 µL of fresh PBS with 2% neutral formalin (cat. HT5011-1CS, Sigma-Aldrich Co., Saint Louis, MO, USA), and subjected to a flow cytometry analysis. At least 40,000 CD3+CD4+ Th cells were collected from each sample.

According to adjusted and previously published protocols [65,66,67,68], we detected the following T-cell subsets: Th1-like (CXCR5—CCR6—CXCR3+CCR4—), Th2-like (CXCR5—CCR6—CXCR3—CCR4+), total Th17-like (CXCR5—CCR6+), total Tfh-like (CXCR5+), classical Th17-like (CXCR5—CCR6+CXCR3—CCR4+), Th17.1-like (CXCR5—CCR6+CXCR3+CCR4—), double-positive Th17-like (CXCR5—CCR6+CXCR3+CCR4+), double-negative Th17-like (CXCR5—CCR6+CXCR3—CCR4—), Tfh1-like (CXCR5+CCR6—CXCR3+), Tfh2-like (CXCR5+CCR6—CXCR3—), Tfh17-like (CXCR5+CCR6+CXCR3—), and double-positive Tfh-like, (CXCR5+CCR6+CXCR3+) cells. The flow cytometry data were analyzed using Kaluza software v2.1 (Beckman Coulter, Indianapolis, IN, USA).

All statistical tests were performed using Statistica 7.0 (StatSoft, Oklahoma, OK, USA) and GraphPad Prism 8 (GraphPad Software, San Diego, CA, USA). All the results are presented as median and interquartile range: Me (25;75). A dispersion analysis was done based on ANOVA statistics. Normality was checked using Pearson’s chi-squared test. The differences between groups were assessed using the nonparametric Mann–Whitney U-test. Significance was set at *p* < 0.05. Correlation analysis was performed using nonparametric Spearman rank test. Significance was set at *p* < 0.05.

Discriminant analysis was used to determine the cytokine profile, which enabled distinguishing patient groups and healthy donors as well as predicting what parameters are mostly common for each group. A forward stepwise analysis enumerating steps, *p*-value significance level, and F-test were performed. A discrimination level was evaluated by Wilks’ lambda. Significance of parameters was determined after drawing scatterplots of canonical values and calculating classification value and Mahalanobis squared distance.

## 5. Conclusions

The obtained results revealed that the cytokine profile varied across different severity levels of COVID-19 and in its influence on progression of disease. Taking into account that some cytokines are characterized by synergistic as well as pleiotropic effects and can mediate cell proliferation and differentiation, their alteration may impact significantly on the long-consequence of COVID-19. Thus, the study of cytokine profiles may provide new insights into the development of the long-consequence of COVID-19. Careful definition of distinct subsets of CD4+ T cells in patients with acute COVID-19 and delineating how cytokines and chemokines imbalance influence T cell responses during the acute phase of infection and SARS-CoV-2-specific memory maintenance in COVID-19 convalescents could reveal novel opportunities for effective COVID-19 treatment in future.

## Figures and Tables

**Table 1:** Plasma levels of cytokines in patients with moderate or severe COVID-19 and healthy donors, Me (25;75).

Parameter, pg/mL	Healthy Donors (*n* = 11)	Moderate COVID-19 (*n* = 41)	Severe COVID-19 (*n* = 32)	Significance, *p*
sCD40L	857 (435;1210)	295 (176;734)	467 (266;1042)	*p*_1,2_ = 0.043
EGF	9.6 (5.5;16.3)	9.0 (6.0;13.1)	12.0 (8.3;18.9)	*p*_2,3_ = 0.013
Eotaxin	57.4 (43.0;74.5)	57.8 (48.5;72.9)	45.6 (36.6;68.2)	*p*_2,3_ = 0.039
FGF-2	41.2 (30.9;45.4)	42.6 (39.8;56.0)	52.7 (42.6;61.1)	*p*_1,3_ = 0.048
FLT-3L	13.4 (9.6;16.7)	13.8 (10.1;19.4)	14.3 (11.3;18.4)	
Fractalkine	103.4 (81.3;161.6)	122.7 (96.4;140.2)	113.3 (89.0;134.6)	
G-CSF	9.2 (5.6;19.0)	43.7 (22.0;74.1)	36.7 (24.3;80.2)	*p*_1,2_ = 0.002 *p*_1,3_ = 0.003
GM-CSF	3.7 (0;5.0)	7.4 (3.5;9.9)	6.3 (3.9;11.3)	
GROa	0 (0;1.8)	5.3 (2.1;10.8)	6.1 (3.1;10.4)	*p*_1,2_ < 0.001 *p*_1,3_ < 0.001
IFN α2	38.5 (36.0;45.6)	54.2 (43.3;65.7)	58.2 (45.6;85.9)	*p*_1,2_ = 0.005 *p*_1,3_ = 0.004
IFN γ	3.1 (2.0;6.3)	10.7 (4.9;17.0)	7.3 (4.3;15.9)	*p*_1,2_ = 0.005 *p*_1,3_ = 0.043
IL-1α	4.1 (3.2;6.8)	5.0 (4.1;8.6)	5.0 (3.2;7.9)	
IL-1β	6.3 (5.3;12.1)	8.4 (6.3;15.6)	10.3 (7.4;14.8)	*p*_1,3_ = 0.043
IL-1RA	2.1 (1.5;3.1)	7.7 (4.3;35.8)	10.9 (3.9;28.1)	*p*_1,2_ < 0.001 *p*_1,3_ < 0.001
IL-2	1.2 (0.9;1.8)	1.3 (1.0;1.9)	1.7 (1.0;2.2)	
IL-3	0 (0;0.7)	0.8 (0;1.1)	0.8 (0.3;1.1)	*p*_1,2_ = 0.013 *p*_1,3_ = 0.009
IL-4	1.2 (0.6;3.3)	1.4 (0.7;2.6)	1.6 (0.6;2.2)	
IL-5	1.9 (1.2;3.0)	3.0 (2.1;4.6)	2.8 (1.8;4.4)	*p*_1,2_ = 0.026 *p*_1,3_ = 0.034
IL-6	0.7 (0.4;1.0)	13.2 (5.2;22.6)	11.2 (1.9;48.1)	*p*_1,2_ < 0.001 *p*_1,3_ < 0.001
IL-7	0.4 (0;0.6)	0.7 (0.4;1.4)	0.8 (0.4;1.4)	*p*_1,3_ = 0.022
IL-8	1.3 (1.1;1.7)	3.8 (2.6;6.6)	5.3 (3.1;7.9)	*p*_1,2_ < 0.001 *p*_1,3_ < 0.001
IL-9	9.9 (6.8;14.6)	12.4 (8.4;21.6)	14.6 (8.8;20.1)	
IL-10	0 (0;0.2)	16.1 (8.3;22.9)	17.0 (6.3;35.1)	*p*_1,2_ < 0.001 *p*_1,3_ < 0.001
IL-12 p40	23.8 (17.9;27.7)	48.8 (36.4;62.1)	37.3 (32.5;62.1)	*p*_1,2_ < 0.001 *p*_1,3_ = 0.002 *p*_2,3_ = 0.049
IL-12 p70	2.5 (1.6;4.0)	1.9 (1.6;3.7)	2.5 (1.8;3.5)	
IL-13	18.7 (10.1;37.3)	21.2 (6.4;36.3)	20.0 (11.6;28.6)	
IL-15	6.5 (5.5;9.3)	15.9 (12.9;22.1)	18.1 (15.7;22.2)	*p*_1,2_ < 0.001 *p*_1,3_ < 0.001
IL-17A	4.9 (4.1;7.4)	6.2 (4.0;8.9)	7.4 (4.9;9.6)	
IL-17E/IL-25	228 (198;313)	326 (257;458)	326 (228;471)	
IL-17F	13.9 (13.4;14.4)	17.0 (14.4;20.2)	17.0 (15.6;21.4)	*p*_1,2_ < 0.001 *p*_1,3_ < 0.001
IL-18	22.0 (9.8;40.0)	89.5 (59.3;127.3)	64.5 (43.6;107.1)	*p*_1,2_ < 0.001 *p*_1,3_ = 0.003
IL-22	0 (0;18.2)	0 (0;21.3)	0 (0;16.4)	
IL-27	580 (548;894)	2208 (1309;2938)	2026 (1467;2941)	*p*_1,2_ < 0.001 *p*_1,3_ < 0.001
IP-10	114 (92;145)	4025 (2153;9499)	8536 (1938;14512)	*p*_1,2_ < 0.001 *p*_1,3_ < 0.001
MCP-1	193 (140;216)	345 (267;462)	340 (251;561)	*p*_1,2_ < 0.001 *p*_1,3_ < 0.001
MCP-3	10.1 (0;17.0)	12.9 (10.1;19.3)	14.2 (10.1;22.3)	
M-CSF	56.4 (40.2;56.4)	528.9 (410.4;786.1)	435.3 (246.2;836.7)	*p*_1,2_ < 0.001 *p*_1,3_ < 0.001
MDC	536 (466;595)	785 (583;843)	629 (503;821)	*p*_1,2_ = 0.009
MIG	720 (636;845)	3402 (1621;4645)	2776 (1618;5375)	*p*_1,2_ < 0.001 *p*_1,3_ < 0.001
MIP-1a	10.1 (7.9;12.9)	13.8 (10.1;16.9)	14.2 (10.1;18.3)	*p*_1,2_ = 0.042 *p*_1,3_ = 0.042
MIP-1b	12.3 (9.6;14.6)	15.8 (13.2;18.9)	14.3 (12.2;19.7)	*p*_1,2_ = 0.015
PDGF-AA	1636 (864;2603)	662 (228;1840)	1375 (502;1831)	*p*_1,2_ = 0.049
PDGF-AB/BB	17870 (9641;21296)	6679 (3165;15740)	11000 (5187;16815)	*p*_1,2_ = 0.016
TGF α	2.7 (1.9;3.1)	3.8 (2.9;5.4)	4.0 (3.1;5.4)	*p*_1,2_ < 0.001 *p*_1,3_ = 0.001
TNF α	13.4 (11.1;20.6)	51.1 (35.7;91.1)	60.0 (40.6;76.7)	*p*_1,2_ < 0.001 *p*_1,3_ < 0.001
TNF β	0.8 (0;2.0)	0.8 (0;2.4)	1.4 (0;3.2)	
VEGF-A	76.6 (46.7;105.1)	58.1 (38.9;122.5)	87.3 (52.1;201.8)	

**Table 2:** Characteristics of healthy donors and patients with COVID-19, Me (25;75).

Parameter with Applicable Reference Values	Healthy Donors (*n* = 11)	Patients with COVID-19	
		Moderate (*n* = 41)	Severe (*n* = 32)
Age	40.0 (32.0;47.0)	61.0 (52.0;69.0) ^	59.0 (49.5;65.0) ^
CT, % lung damage		32 (24;44) *	60 (48;70) *
CRP, mg/L (0–5.0)		50.1 (18.1;73.3) **	72.5 (40.9;109.5) **
D-dimer, µg/mL (0–5.0)		0.55 (0.33;1.25)	0.62 (0.40;1.44)
Ferritin, ng/mL (30–400)		318.2 (187.5;562.2)	422.6 (150.2;713.4)
Fibrinogen, g/L (1.9–4.3)		5.8 (4.5;6.4)	6.4 (4.9;6.9)
ALT, U/L (0–41)		34.0 (19.5;66.2)	34.1 (23.9;59.0)
AST, U/L (0–40)		35.4 (27.7;59.0)	38.7 (32.3;58.4)
LDH, U/L (135–225)		300 (232;343) *	361 (298;415) *
Haemoglobin, g/L	140.0 (137.0;151.0)	136.4 (123.0;145.1)	141.5 (132.2;148.6)
RBC, ×10^12^	5.00 (4.77;5.13)	4.72 (4.12;4.99) ^^	4.73 (4.48;4.91) ^^
Ht, %	43.9 (42.3;46.2)	39.2 (35.3;41.8) ^	40.2 (38.5;42.5) ^
Plt, ×10^9^	207 (157;235)	187 (151;229)	197 (147;301)
WBC, ×10^9^	6.1 (5.4;7.8)	5.0 (3.8;7.0)	4.8 (3.9;9.0)
Lymphocytes, ×10^9^	1.83 (1.64;2.06)	1.17 (0.92;1.46) ^	1.02 (0.74;1.31) ^
Monocytes, ×10^9^	0.58 (0.39;0.74)	0.50 (0.30;0.57)	0.41 (0.27;0.53) ^^
Neutrophils, ×10^9^	3.55 (2.91;4.56)	3.29 (2.37;4.21)	3.30 (2.66;7.70)
Eosinophils, ×10^9^	0.14 (0.08;0.28)	0.03 (0.01;0.06) ^	0.01 (0;0.04) ^
Basophils, ×10^9^	0.07 (0.06;0.09)	0.02 (0.01;0.04) ^	0.01 (0.01;0.04) ^
Neutrophils/Lymphocytes	1.76 (1.52;2.32)	2.82 (1.99;3.89) ^	3.51 (2.26;5.46) ^

Abbreviations: ALT—alanine aminotransferase, AST—aspartate aminotransferase, CRP—C-reactive protein, CT—computer tomography, Ht—hematocrit, LDH—lactate dehydrogenase, Plt—platelets, RBC—red blood cells. Cut-off values are given for an appropriate laboratory. * *p* < 0.01, compared severe and moderate COVID-19; ** *p* < 0.05, compared severe and moderate COVID-19; ^ *p* < 0.01, compared with HD; ^^ *p* < 0.05, compared with HD.

## Data Availability

Not applicable.
